# Beyond journal impact factors?

**DOI:** 10.1186/1478-811X-8-4

**Published:** 2010-03-10

**Authors:** Stephan M Feller

**Affiliations:** 1 Editor in Chief

## Editorial

CELL COMMUNICATION AND SIGNALING, which is published since August 2008 as the official journal of the Signal Transduction Society http://www.sigtrans.de has recently been included in the impact factor tracking system of Thomson Reuters ISI. For a relatively new enterprise like ours, this appears to be an important step towards establishing ourselves in the realm of serious scientific journals.

Nowadays, many scientists will simply not publish in a journal that does not have an impact factor. There are multiple reasons for this. One is that many universities have started to distribute funds according to formulas that are directly linked to the number of publications a researcher produces and the impact factors of the publishing journals. Therefore, getting a paper into a certain journal and publishing two short papers rather than one longer one (with the same data) can have a major impact on the financial viability of a research group or department. Whether this increasing dependency of researchers on journal impact factors has a positive impact on the speed and quality of their research and of the resulting publications, i.e. their public visibility and the actual output of data and whether it is beneficial to science and society in general, is at least somewhat doubtful, as I shall detail further below.

It has become a way of life for many researchers to create, for each emerging manuscript, a list of possibly suitable journals, which are ranked strictly according to their impact factors. Submission of the manuscript then starts at the top of the list. Quite often even the authors know that the chances for acceptance of their work in this top-tier journal are minimal, but nevertheless 'one might get very lucky', or 'one might at least get a foot in the door' (i.e. a chance to resubmit after a very major revision), or 'one might get good suggestions for further experiments from the reviewers', or... In reality, however, this strategy almost always leads to multiple rejections in a row, while numerous hours are spent on reviewing, reformatting and rewriting the manuscript, leading to a substantial loss in productive research time for both authors and reviewers.

A second reason for the prominent role of journal impact factors is that they are used in an ever-growing number of career-deciding evaluations by review boards of funding agencies, recruitment committees, university governing bodies, external advisory panels, governmental research assessments etc. Especially researchers in the early stages of their career tend to greatly depend on the outcomes of these evaluations. Of course, with an ever-increasing number of evaluations taking place, the commission/committee members neither have the time nor, in some cases, the qualifications, to read and digest all relevant publications properly. Instead, they are more and more tempted to simply look at journal impact factors as a quick, if inappropriate, substitute for a proper review.

In addition, the inflated dependency on journal impact factors and hence the need for manuscript acceptance in a specific journal can have detrimental consequences for scientists that are purely driven by commercial interests. As one colleague told me not too long ago, it took him nearly three times as long to get the manuscript into its final, formally-correct shape after the scientific review was completed than it took him to generate the data in the first place. At the end he 'was ready to strangle the editor with his bare hands' since the editor seemed to have no interest in the actual work and only wanted the formal regulations like word number and figure size limits of the journal to be strictly met. This may have just been an inexperienced editor, but the same can happen if costly print journals are under pressure to maximise their profits, and it is not helping scientists and society at all. When asked why he put up with this, my colleague's answer was, not surprisingly, 'I need the journal's impact factor for my next application'.

Along the same lines, figures that are cropped to a degree that makes it impossible to judge data quality, or that are composed of a dozen down-scaled and now stamp-sized pictures that have lost much of their initial high resolution, are not an uncommon phenomenon these days, even in high impact print journals. It may be fun to joke about this in a lab's journal club, but it does not advance science.

Scientists are increasingly forced into becoming once again hunters and gatherers, this time of 'impact points', which are in some way (this of course differs from place to place) calculated from journal impact factors. This can have some rather ugly consequences that deserve critical discussion. Only two will be touched upon here:

While in the past some senior scientists have forced their names onto papers even if they had contributed little to nothing just to cuddle their egos, now there is a second, very compelling reason for them to do so even more often: impact points = research money.

Another creature living in the researchers' version of Pandora's box that is raising its ugly head even further these days thanks to the growing impact point-mania, is the spectre of data fabrication. If impact points become the primary, overriding factor deciding on tenure or termination of a researcher's career, it becomes ever more likely that the potentially threatened researchers will be under so much pressure that they begin to lose their integrity and decide to fabricate data - just this once, just one figure, just to appease that one reviewer and get their paper into that one particular journal, and then just once more to get that big grant, and then...

So what does the future hold? I think there may be hope for things to evolve towards a more balanced and productive way of sharing scientific research findings and evaluating their significance. We are currently in the midst of a major revolution in scientific publishing. Many restricted access print journals may not realise it yet, but they are on their way out. Increasingly, research institutes close down their print journal libraries as soon as they need more space and/or the librarian is due for retirement. A new generation of researchers has grown up which uses the internet as an integral part of their daily lives and cannot be bothered to leaf through print journals, and a rising number of scientists is growing tired of not being able to freely access research papers that have been generated with public funds.

With the rise of open access online journals that lack a mandatory print version and are freely available around the world, the journal as such loses some of its status as a strictly defined entity. A 'pure' online journal does by and large not need regular issues or deadlines and it does not have space limitations that prevent it from publishing important data or manuscripts. It becomes in many ways primarily a humble, community-serving hub for organising the peer-review and subsequent distribution of qualifying scholarly information, and that is what it really should be. If all information is readily available with a couple of mouse clicks on PubMed and similar sites, suddenly it does not seem to matter so much anymore to which journal it is linked and the actual paper and its data become more important once more.

Clearly, it does not make any sense to go back to the ways of the pre-journal publishing era, i.e. publishing without a proper peer review. It may, however, be useful if all journals decided to post all versions of a submitted paper, as well as (anonymously) the reviewers' and editors' comments. Authors would then have to think more carefully about whether their work is actually ready to be submitted, instead of overloading the system with poorly drafted 'exploratory' manuscript versions. Editors would have to select their reviewers more carefully for competence and fairness and reviewers may have to hold back somewhat on unfair comments and tactics that are intended to slow down publications of competitors.

With the potentially diminishing importance of journal impact factors, what are the chances for eventually replacing them all together by article-specific impact factors? This is a difficult issue. Download numbers, although easy to establish, are certainly no proper measure for data quality and significance. More important in practical terms, journal impact factors are currently in use as the scientific communities' crystal balls to estimate the significance of a manuscript that has just been published. It is unlikely that this seemingly convenient prediction tool, albeit clearly imperfect, will go completely out of fashion any time soon. Analysing citation numbers (with self-citations subtracted?) will only work after some years and has limits as well. Nevertheless, it would be good to calculate article-specific impact factors, even if they would sometimes be negative, for example for papers that never get cited by anybody but the authors themselves, and certainly for retracted papers. If this was done, say 2, 5, 10 and 20 years after publication of a paper, it should give some robust information about which articles have a real impact on scientific research. By incorporating this information into databases like PubMed, authors would get credit for making a substantial contribution (or not), and more volatile 'bets' on the future relevance of a paper based on journal impact factors should become diminished in their perceived importance. At the end of the day, scientists may learn to love this new way of evaluating their significance, since it could take away some of the hype and lead to a better balance between speculative short-term praise and proper long-term credit based on facts, allowing them to focus once more on conducting their research.

